# Environmental and trophic determinism of fruit abscission and outlook with climate change in tropical regions

**DOI:** 10.1002/pei3.10011

**Published:** 2020-04-22

**Authors:** Sébastien Tisné, Marie Denis, Hubert Domonhédo, Benoît Pallas, Michel Cazemajor, Timothy J. Tranbarger, Fabienne Morcillo

**Affiliations:** ^1^ Cirad UMR AGAP Montpellier France; ^2^ AGAP Univ Montpellier CIRAD INRA Montpellier SupAgro Montpellier France; ^3^ CRA‐PP/INRAB République du Bénin; ^4^ PalmElit SAS Montferrier‐sur‐Lez France; ^5^ IRD Univ Montpellier DIADE Montpellier France; ^6^ Cirad UMR DIADE Montpellier France

**Keywords:** climate change, *Elaeis guineensis*, environmental regulation, fruit abscission, multivariable models

## Abstract

Fruit abscission facilitates the optimal conditions and timing of seed dispersal. Environmental regulation of tropical fruit abscission has received little attention, even though climate change may have its strongest impacts in tropical regions. In this study, oil palm fruit abscission was monitored during multiple years in the Benin Republic to take advantage of the climatic seasonality and the continuous fruit production by this species. An innovative multivariable statistical method was used to identify the best predictors of fruit abscission among a set of climate and ecophysiological variables, and the stage of inflorescence and fruit bunch development when the variables are perceived. The effects of climate scenarios on fruit abscission were then predicted based on the calibrated model. We found complex regulation takes place at specific stages of inflorescence and bunch development, even long before the fruit abscission zone is competent to execute abscission. Among the predictors selected, temperature variations during inflorescence and fruit bunch development are major determinants of the fruit abscission process. Furthermore, the timing of ripe fruit drop is determined by temperature in combination with the trophic status. Finally, climate simulations revealed that the abscission process is robust and is more affected by seasonal variations than by extreme scenarios. Our investigations highlighted the central function of the abscission zone as the sensor of environmental signals during reproductive development. Coupling ecophysiological and statistical modeling was an efficient approach to disentangle this complex environmental regulation.

## 1 INTRODUCTION

Climate change is predicted to result in hazardous weather events with detrimental effects on food production systems especially in tropical countries (Brown & Funk, 2008; Myers et al., 2017). Under warmer, colder, dryer, or wetter growth conditions, plants activate adaptive mechanisms to ensure species survival during both seasonal and permanent changes in the environment. One key plant adaptation process that is particularly sensitive to environmental conditions is organ abscission, by which a plant eliminates whole organs such as leaves, flowers, floral organs, and fruit (Addicott, 1982; Patharkar & Walker, 2016; Sawicki, Ait, Clement, Vaillant‐Gaveau, & Jacquard, 2015). The most obvious and best known example of environmental effects on organ abscission in temperate regions is the seasonal loss of leaves in autumn, which is modulated by changes in photoperiod, temperature, and water availability, and considered to be an adaptive mechanism to optimize plant carbon and nitrogen homeostasis and to reduce the risk of freezing (Addicott, 1982). A meta‐analysis of observations recorded from 1931 to 2010, showed that, in the Northern Hemisphere, leaf senescence and abscission of deciduous trees are delayed in response to an increase in temperature, suggesting the timing of organ abscission could be altered by global environmental changes (Gill et al., 2015).

Fruit abscission may be particularly sensitive to changes in environmental conditions, given that this developmentally regulated process must occur under the appropriate conditions and at the right time to ultimately allow seed dispersal and species survival. If fruit are shed prematurely, that is, before seed and embryo maturation are complete, or too late in relation to seasonal climate changes, reproductive success may be jeopardized. While it is clear that environmental factors have consequences for organ loss, it is not yet clear at which stages in the abscission process the environmental variables are sensed. The separation of an organ from the plant body takes place in cells in a specialized abscission zone (AZ). Based on studies using model plants including *Arabidopsis thaliana* and tomato, the process of organ abscission can be divided into four main steps: (a) the differentiation of AZ cells, which eventually (b) acquire the competence to respond to abscission signals and initiate cellular responses, which in due course, lead to (c) cell wall remodeling and cell wall degradation, and result in cell separation between adjacent AZ cell layers and fruit drop, and finally, (d) the transdifferentiation of a protective layer on the proximal side of the AZ (Aalen, Wildhagen, Sto, & Butenko, 2013; Estornell, Agusti, Merelo, Talon, & Tadeo, 2013; Meir et al., 2019). During this highly coordinated sequence of events, the cellular responses in the AZ include changes to molecular, metabolic, and structural cell components. However, environmental factors that can initiate the abscission process and control abscission execution, which includes the activation of signaling and regulatory factors that control the expression of cell wall hydrolytic enzymes, are not well known, and to date, studies have focused on only a few crop and model species such as apple, tomato, citrus, and Arabidopsis (Botton et al., 2011; Estornell et al., 2013; Meir et al., 2010; Roberts, Elliott, & Gonzalez‐Carranza, 2002). Interestingly, a recent molecular study on drought‐induced leaf abscission in *Arabidopsis* indicated that the signaling pathways are similar to those identified in *Arabidopsis* floral organ abscission (Patharkar & Walker, 2016). However, studies on how the environment affects organ abscission of perennial crops is complicated by the size of the plants and by the difficulty involved in growing them in controlled conditions, and so whether the same pathways function in all abscission systems remains to be determined (Yu, Hu, Doust, & Kellogg, 2019). One way to circumvent the problem of controlled environmental conditions is to perform studies which take advantage of natural seasonal variations that occur over a period of years.

The African oil palm *Elaeis guineensis* grows in tropical regions with strong seasonality that could increase with climate change and cause more heat and drought stresses in these regions (Paterson, Kumar, Shabani, & Lima, 2017). *Elaeis guineensis* has an indeterminate growth pattern and produces fruit bunches throughout the year, up to two per month, whose variability depends on the environmental conditions, particularly water availability (Legros et al., 2009; Oettli, Behera, & Yamagata, 2018). Oil palm is cultivated for the large quantities of lipids synthesized and stored in both the mesocarp and endosperm tissues (Dussert et al., 2013; Tranbarger et al., 2011). On average, bunches are harvested 6 months after anthesis, when the first fruits detach and fall to the ground. Histological analysis revealed a very large primary AZ between the fruit and the pedicel of the oil palm, in addition to adjacent AZs where cell separation occurs only after separation in the primary AZ (Henderson & Osborne, 1990; Roongsattham et al., 2016). Like in other fruit species, the primary AZ differentiates in the pre‐anthesis inflorescence before fruit development begins (Henderson & Osborne, 1990). The primary AZ acquires the competence to activate and execute abscission only during the later stages of fruit ripening, and is highly dependent on the level of ethylene endogenously synthesized by the fruits, and enhanced by exogenously applied ethylene (Henderson & Osborne, 1990; Roongsattham et al., 2012, 2016).

In the present study, we took advantage of the contrasted seasons that occur in the region of Benin Republic where a self‐pollinated population of oil palm is cultivated and manually pollinated. We used the date of ripe bunch harvest to record the days to natural fruit drop (DFD) over a series of years. In parallel, we used an in vitro test to phenotype the competence to execute abscission in the fruit AZ in the same population. We combined recorded climate and phenotypic data to determine which environmental variables affect fruit abscission in this species, and at what stages of inflorescence/bunch development these environmental factors are perceived. A model combining statistical and ecophysiological approaches was calibrated and used to predict the impacts of possible future climatic scenarios on DFD in relation with the intensity of cell separation in the fruit AZ.

## 2 MATERIALS AND METHODS

### 2.1 Plant material

The study was carried out in an experimental plantation of the *Centre de Recherches Agricoles‐Plantes Pérennes* (CRA‐PP), Pobè, Benin Republic, on single plot at WGS 84:6.971808° N 2.678713°E. The soil of the plot is a hydromorphic sandy beige type, fertilized with an average of 1 kg of urea and 2 kg of KCl per palm per year. In addition, recycling of pruned fronds was practiced in the plot to improve soil fertility as described previously (Aholoukpé et al., 2016). Plant material was derived from a self‐pollinated individual (DA115D) which originated from the Deli population. Three individuals from DA115D were then self‐fertilized to produce three populations, containing 50, 46, and 42 individuals, respectively, planted between 2000 and 2005 with a density of 143 trees/ha (Figure S1). The plants were between 10 and 15 years old at the start of the study. For each individual, the appearance of all female inflorescences was recorded and at anthesis, each inflorescence was labeled and manually pollinated. The pollination date was recorded and the bunch resulting from each pollinated inflorescence was then monitored up to harvest, which began after the first detached fruits were observed on the ground beneath the bunch.

### 2.2 Phenotyping fruit abscission traits

The common practice to decide when to harvest ripe oil palm fruit bunches in plantations is for workers to observe three to five detached fruits on the ground under a palm tree (Corley & Tinker, 2016). In the present study, we used the recorded date of harvest based on this method to indicate the DFD. The bunch weight (BW) and DFD were recorded between June 2015 and April 2018. It included the BW and DFD data for 673 ripe bunches. During the same period, the values of an abscission index (AI), which assesses the competence of the fruit AZ to execute cell separation at the base of the oil palm fruit was recorded for 493 bunches. The AI was calculated based on a previously described in vitro phenotype test, with modifications (Fooyontphanich et al., 2016; Tranbarger et al., 2019). In this study, the test was applied to fruits collected from bunches 160 days after pollination (DAP), plus or minus 2 days. This ripeness period was chosen to perform the tests on pre‐abscission fruit because at this stage, DFD had not yet occurred in 92% of the bunches (Figure S2). From the upper middle section of each bunch, 8–12 fruit spikelets were removed. From these spikelets, approximately 24 similar fruit were selected and the base of each fruit containing the AZs was then sliced longitudinally to obtain at least two approximately 1–1.5 mm thick slices. This meant each slice contained both the large primary AZ and the adjacent AZs previously described (Henderson & Osborne, 1990; Roongsattham et al., 2016). A total of 45 slices of fruit base were collected from each bunch and placed on Petri plates with a dampened Whatman filter paper. The fruit bases containing the non‐separated AZs were then incubated at room temperature for 22 hr, after which forceps were used to apply pressure by twisting the slices to test for separation at both the primary and secondary AZs. For the phenotype, four classes were defined and attributed to each fruit slice tested: A, no cell separation; B, partial cell separation in primary AZ only; C, extensive cell separation in primary AZ only; and D, complete cell separation in both primary and adjacent AZs (Figure S3). In the present study, we used the following formula to calculate AI:

$$AI=\frac{3*n_{A}+1*n_{B}+\left(-1\right)*n_{C}+\left(-3\right)*n_{D}}{n_{A}+n_{B}+n_{C}+n_{D}},$$

where *n* is the total number of observations per class. Positive and negative values of AI reflect low and high level of competence of the fruit AZ to execute cell separation, respectively.

### 2.3 Climate data acquisition

The climatic data maximum temperature (Tmax ,°C), minimum temperature (Tmin, °C), relative air humidity (RH, %), rainfall (R, mm), solar radiation (SR, cal.cm^‐2^.d^‐1^), and evaporation (mm) were recorded daily manually at the agroclimatic station located at Pobè. SR was measured using an alcohol actinometer. Daily potential evapotranspiration was estimated with a Piche evaporimeter (mm). The RH at 8, 13, and 18 hr was calculated from observations of the dry and wet bulb thermometers (Walter, 1942).

### 2.4 Calculation of ecophysiological variables

Five ecophysiological variables were calculated using climate and individual production data: three environmental variables including the maximum daily vapor pressure deficit (VPD), the fraction of transpirable soil water (FTSW), and the cumulative thermal time (TT) and, two trophic variables, the supply–demand ratio (SD) and the daily reproductive demand (DRD).

The VPD was estimated from daily Tmax and minimum relative humidity following the method previously described (Allen, Pereira, Raes, & Smith, 1998). A simplified water balance model (Pallas et al., 2013) was used for the daily estimation of water deficit intensity estimated with the FTSW (Sinclair & Ludlow, 1986). The FTSW scale ranges from 0 to 1, where low values reflect water deficit conditions. The model (a) uses an estimation of the daily evaporative demand as the product of potential evapotranspiration and crop coefficient (0.7, Combres et al., 2013), (b) includes transpiration limitations due to stomatal closure (decrease in transpiration when FTSW <0.4) as described in Legros et al. (2009), and (c) assumes a crop coefficient equal to 1 (Allen et al., 1998). The total transpirable soil water content was set at 200 mm, which is the typical value for oil palm cultivation (Dufrêne, 1989). The daily TT was estimated from daily Tmin and Tmax using a trapezoid response curve with four cardinal temperatures (Tbase = 11°C, Topt1 = 26°C, Topt2 = 30°C, and Tlim = 48°C) as previously described (Combres et al., 2013; Figure S4). TT was calculated by the sum of daily TT values throughout inflorescence/bunch development.

The method used to estimate the daily carbon SD at the tree scale is described in Pallas et al. (2013). Following this method, the carbon demand was split into vegetative and reproductive demand. The DRD depends on the number of developing bunches and fruit and their phenological stages on the tree on a given day. The growth of the structural part of the bunches was assumed to occur over a period of 300 days before harvest with a constant demand equal to the product of their mean final weight (1,500 gDM) and their chemical cost (1.4 gC gDM^‐1^) divided by the length of the growth period. Fruit growth was assumed to occur during the 180 days preceding harvest, and lipid biosynthesis during the last 90 days. The calculation of the growth demand before and during lipid biosynthesis included (a) the mesocarp/kernel ratio (2.3), (b) the mean oil contents of the mesocarp (80%) and kernel (52%), and (c) the chemical cost of the fruit compartment with (3.2 gC gDM^‐1^) or without oil (1.4 gC gDM^‐1^), and the mean observed fruit dry weight (6.4 gDM). The vegetative part was considered as a bulk compartment with a constant daily demand of 5.3 gDM/day and corresponding to the situation in which 10 leaves are expanding concomitantly at a given date. The supply of assimilates was computed by multiplying (a) the daily radiation interception efficiency, (b) the potential radiation use efficiency, (c) a “stress” coefficient estimated from the water balance, and (d) the photosynthetically active radiation at the experimental site. Photosynthetically active radiation was estimated as the product of solar radiation provided by the weather station and a climatic efficiency equal to 0.48. The radiation interception efficiency was modeled based on the Beer–Lambert law assuming a constant leaf area index of 4 and an extinction coefficient of 0.8. The radiation use efficiency (4.6 gDM/MJ) was also assumed to be constant throughout the experimental period.

### 2.5 Statistical analyses

Two approaches were used to analyze the effect of environmental and trophic determinism on fruit traits to identify which variable had an effect, and at which stages of inflorescence and bunch development. A three‐day grid of times from −180 (individualization of the floral meristem) to +180 DAP (ripe fruit) was used to calculate either the average values over three days (Tmax, Tmin, RH, VPD, FTSW, DRD, and SD) or the cumulative values over 15 days (R and SR) of each variable. Nine matrices associated with each variable were then constructed. Within each matrix the i^th^ row corresponds to each bunch analyzed and the j^th^ column corresponds to the value of the corresponding climatic/ecophysiological variable at the j^th^ time for each bunch.

In the first approach, univariate linear regression models were fitted with abscission traits as the dependent variable and each climatic/ecophysiological variable for each time (*n* = 9*121 = 1,089) as the explanatory variable. The *p*‐value profiles were examined to identify associations between the environment and trophic status, and the abscission traits. Regression models were fitted using the lm function in R statistical software (R Core Team, 2018).

In the multivariable analyses, a group penalized approach was used to identify environmental/trophic variables as well as their associated times. This kind of approach has been used in genetic studies and produced promising results (Ayers & Cordell, 2010; Denis et al., 2014; Huang & Breheny, 2012). To select the environmental/trophic matrix, called group, as well as relevant times within the matrices, a bilevel selection that uses a composite minimax concave penalty (composite MCP, Breheny & Huang, 2009) was used. The selection was guided by a penalty parameter, which was selected by performing a 10‐fold cross validation. The R package grpreg was used for these analyses (Breheny & Huang, 2009, 2015).

### 2.6 Climate change simulations and prediction of its effect on the fruit abscission traits

Different hypothetical climate scenarios were simulated to evaluate the potential impact of climate change on the abscission traits DFD and AI. These scenarios were built using meteorological data recorded at the study site from 2014 until 2018. For the sensitivity analysis, three different algorithms were used to modify the daily Tmin and Tmax, and rainfall frequency (RF) and intensity (RI). In the simulated meteorological database, the average daily temperature (T) increase ranged from 0°C to 4°C. Modifying RF consisted in removing on average of 0% to 50% of the rainfall events, while modifying rainfall intensity consisted in multiplying daily rainfall intensity by an average value ranging from 0.5 to 1.5. All the combinations of these variations were considered concomitantly, representing 150 scenarios, and each scenario was replicated 10 times. Ten replicates were used to simulate randomness in the climate impact scenarios. Resulting daily values were thus simulated assuming normal distribution for increased temperatures and rainfall intensity, with variances equal to half the value of the average effect. Removal of rainfall events due to changes in RF was simulated with a Bernoulli law.

For each simulation day, the relative humidity value was computed from a linear model including Tmin and Tmax, and rainfall. Similarly, evapotranspiration was estimated from a linear model including Tmin and Tmax, rainfall, and radiation. The VPD of relative humidity and temperatures was estimated using equations described in Allen et al. (1998). FTSW was calculated using the balance water model with the simulated evapotranspiration values. Stress coefficients computed from the water balance and solar radiation were subsequently used to estimate new values of SD.

All these simulations led to 1,500 simulated environmental matrices based on the environmental data recorded. In the analysis of each scenario, this made it possible to predict the abscission traits for each bunch recorded based on the average value of coefficients obtained with the group penalized method. The statistical significance of the T, RI, and RF effects on predicted fruit trait values were assessed using Spearman correlation coefficients between T, RI, and RF values for each scenario, and the annual or monthly average values of predicted fruit traits.

## 3 RESULTS

### 3.1 Oil palm fruit abscission is subject to annual seasonal cycles in a tropical climate

**Figure 1:**
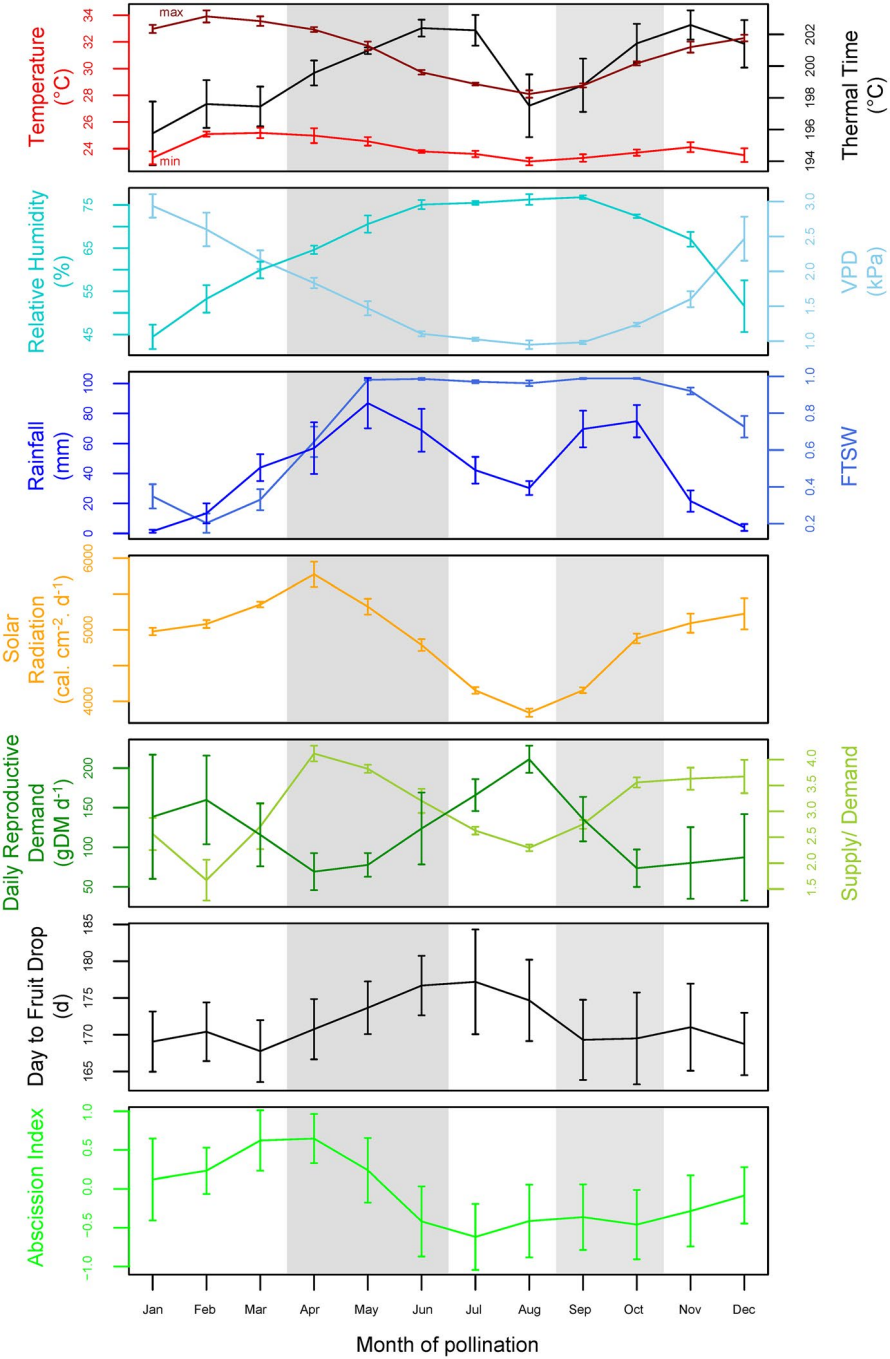
Variation in environmental and fruit abscission traits in oil palm grown in Pobè, Benin. Variations in the 10 climate/ecophysiological variables and 2 fruit abscission traits are plotted against the months when artificial pollination was performed. Each of the four contrasted seasons is indicated by shading. Vertical bars represent interannual standard deviation for the environments and both interannual and bunch standard deviation for abscission traits. Tmax, maximum temperature (°C); Tmin, minimum temperature (°C); RH, relative humidity (%); R: rainfall (mm); SR: solar radiation (cal.cm^‐2^.d^‐1^); TT: cumulative thermal time (°C), VPD: maximum daily vapor pressure deficit (kPa), FTSW: fraction of transpirable soil water, DRD: daily reproductive demand (g), SD, supply–demand ratio; DFD, days to fruit drop; AI, abscission index. The plants were between 10 and 15 years old at the start of the study

The climate in Pobè (Benin), during the study period, showed distinct annual patterns with four contrasted seasons: a long dry season lasting from November to March with warm dry air and high radiation, a long rainy season from April to June with warm moist air and high radiation which decreased progressively, a short dry season in July and August with colder moist air and low radiation, followed by a short rainy season in September and October with colder moist air with progressively increasing radiation (Figure 1, Figure S5). The monthly average climatic variables recorded over a 12‐year period showed that seasonal variations were high, while interannual variations in all variables except rainfall were moderate (Figure S5). The ecophysiological variables (i.e., TT, VPD, FTSW, DRD, and SD) calculated for the period 2014–2018 also showed intra‐annual variations (Figure 1). Surprisingly, unlike the trophic variables DRD and SD, the variations in VPD and FTSW did not appear to be associated with the alternation of dry and wet seasons (Figure 1). Indeed, the short dry season had a high relative humidity, which creates a weak evaporative demand and maintains a high FTSW, in contrast with the long dry season, which has a strong evaporative demand and a lower FTSW.

In the 2015–2018 period, the fruit abscission traits (i.e., DFD and AI) of each individual in the same progeny were recorded (Figure 1). The DFD ranged from a monthly average of 167–177 DAP during this 4‐year period, with the longest DFD recorded for bunches pollinated in June–July (Figure 1b). Fruit drop observed over a 12‐year period in the population studied revealed similar seasonal variation, with a variation in monthly average of up to 17 days (Figure S5). The AI, which allowed us to phenotype the competence of the fruit AZ to execute abscission, was also subject to seasonal variations, with the highest values recorded for bunches pollinated at the end of the long dry season in March and April (Figure 1). This peak AI corresponds to fruit that would usually drop during the short rainy season (September to October; Figure S6). In contrast, the lowest AI values were observed in July at the end of the longest rainy season, which corresponds to fruit that would drop in December, at the beginning of the long dry season (Figure 1).

### 3.2 Identification of the climatic and ecophysiological determinism of fruit abscission traits using a group penalized method

In our first univariate approach, we tested the environmental and trophic effects on DFD and AI individually using a sliding three‐day window during the inflorescence and bunch development period, when the individualization of the floral meristem (approximately −180 DAP, Adam et al., 2011) and fruit bunch maturity (at around + 180 DAP) occur. Traces of *p*‐values indicated that all variables had significant effects which varied depending on the phenological stage and the abscission trait examined (Figure S7). We used a multivariable approach to identify the variables with a direct effect, and to determine at which inflorescence/bunch developmental stages these effects were perceived. We chose a group penalized method that enables simultaneous analysis of all covariates by selecting and estimating regression coefficients.

**Figure 2:**
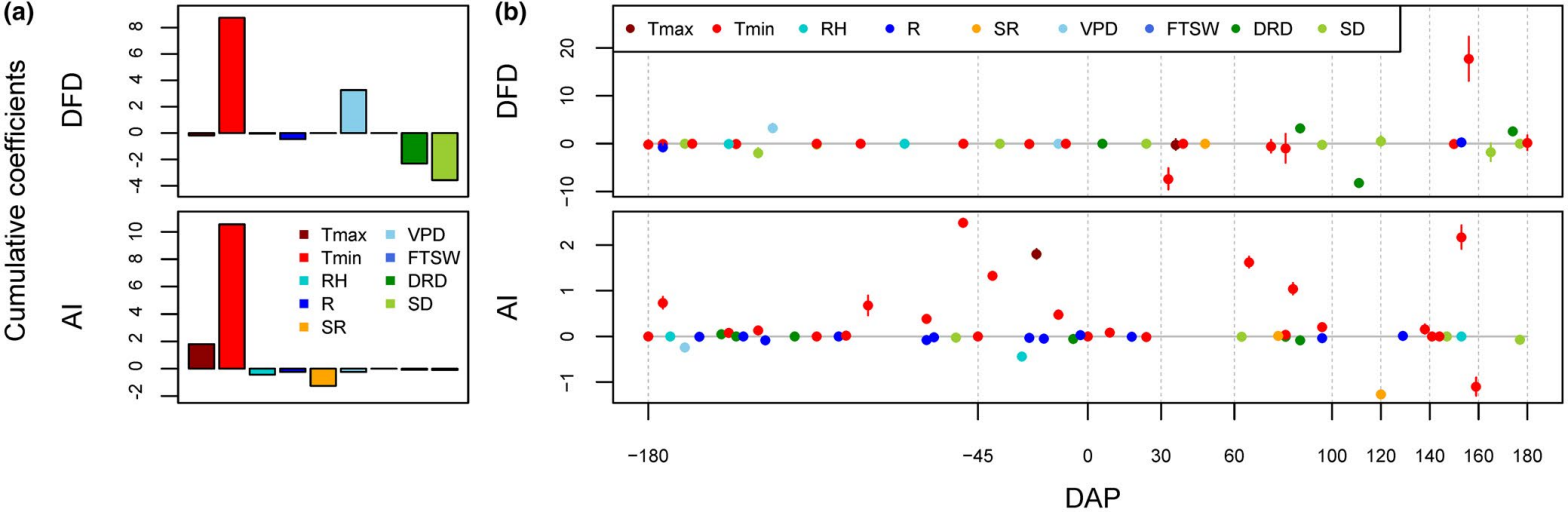
Environmental and trophic determinism of fruit abscission traits in oil palm. Cumulative coefficients (a) and patterns of coefficients (b) obtained for 9 environmental/trophic variables using a penalized method are plotted for the three fruit traits. Known stage transitions during inflorescence/bunch development are indicated by dotted vertical lines on the corresponding days after pollination. Tmax, maximum temperature; Tmin, minimum temperature; RH, relative humidity; R: rainfall; SR: solar radiation; VPD: maximum daily vapor pressure deficit, FTSW: fraction of transpirable soil water, DRD: daily reproductive demand, SD, supply–demand ratio; DFD, days to fruit drop; AI, abscission index

Coefficients estimated throughout floral and bunch development revealed distinct determinism for the two abscission traits (Figure 2a and 2b). The cumulative coefficients over the whole development period showed that Tmin was the major determinant of DFD and AI (Figure 2a). VPD, DRD, and SD had specific but weaker effects on DFD, while Tmax, SR and RH only affected AI (Figure 2a).

Each selected variable displayed a distinct pattern of effect depending on the developmental stage examined (Figure 2b). For AI, Tmin had effects throughout floral and fruit development with peaks at around −45, 70, and 150–160 DAP. Positive (−45 and 70 DAP) and negative (160 DAP) relationships between Tmin and AI at these three specific stages were found in univariate analysis (Figure S7). DFD was affected by Tmin during fruit development at around 30 DAP and then strongly at around 150 DAP in the same period as already observed with the AI (Figure 2b). Interestingly, a higher Tmin at an early stage of fruit development decreased DFD, but conversely increased DFD of ripe fruits (Figure 2b, Figure S7). In addition to these shared effects of Tmin, specific environmental/trophic variables were also selected depending on the abscission trait concerned (Figure 2b). The environmental variables affect both traits: Tmax (at −20 DAP), RH (at −20 DAP), and SR (at 120 DAP) had effects on AI values, while VPD affected DFD (at 130 DAP). In contrast, the trophic variables (DRD and SD) affected only DFD sporadically after 60 DAP (Figure 2b).

The cumulative TT was calculated to assess whether the recurrent effects observed for temperature reflected an effect of TT on the abscission traits. The TT at 160 DAP was not correlated with the DFD (*r* = −.003, *p*‐value = .93), while with AI, it was (*r* = −.14, *p*‐value = .002, Figure S8). However, the group penalized analysis performed at a TT scale showed that the raw temperatures were still the major determinants of AI, with similar effects found at the same developmental stages, consistent with those found with the analysis based on the DAP time scale (Figure 2b).

### 3.3 Prediction of the effect of climate change on traits related to fruit abscission in oil palm

**Figure 3:**
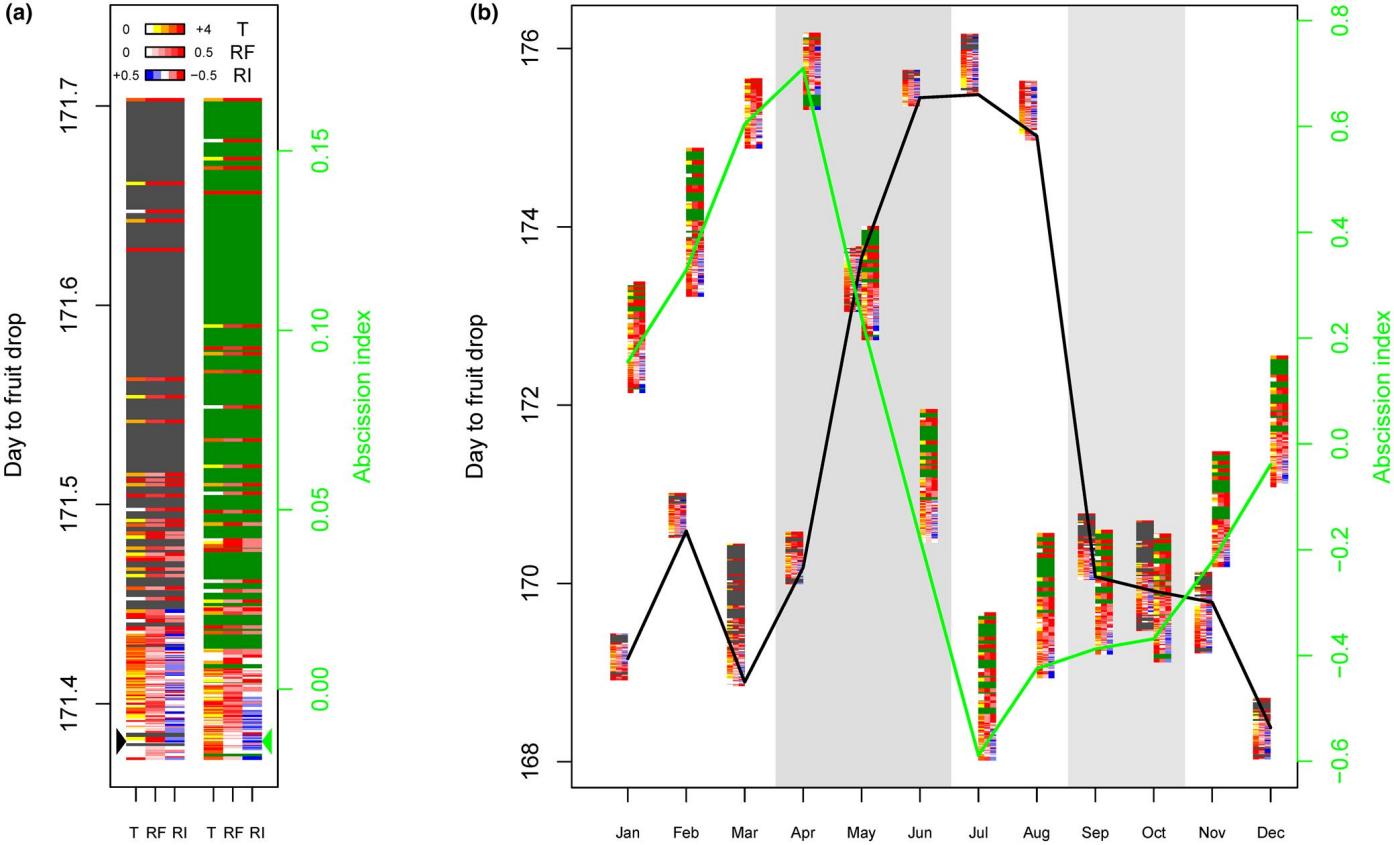
Predicted impact of climate scenarios on fruit abscission traits in oil palm. Annual (a) and monthly (b) average predicted values of DFD (gray) and AI (green) are plotted for all 150 climate scenarios simulated using all possible combinations of changes in temperature (T), rainfall frequency (RF), and rainfall intensity (RI), each scenario being represented by a line with three colors corresponding to environment variables. The color scale indicates the intensity and direction of the variation of the corresponding environmental variables. The arrows (a) and the solid lines (b) indicate the climatic scenario corresponding to current environmental conditions. Each of the four contrasted seasons is indicated by shading

Model fitting quality measured by the correlation between observed and predicted values was 0.63 and 0.69 for DFD and AI, respectively (Figure S9), which should enable acceptable prediction of variations in fruit abscission caused by climate change. Combinations of variations in temperature (T), RF, and RI were simulated leading to 150 possible climate change scenarios in the region, and their effects on abscission traits were predicted. Most of the scenarios analyzed increased the annual average of both DFD and AI (Figure 3a). However, the range of the annual average DFD and AI obtained with climate simulations was lower than the range found for current seasonal variations (0.3 vs. 8 DAP for DFD, and 0.18 vs. 1.4 for AI, Figure 3). The combination of increased temperature and reduced RF and intensity predicted the highest average annual DFD (Figure 3a, Table S1). In contrast, only the combination of a decrease in both RF (ρ = 0.66) and intensity (ρ = 0.65) predicted the highest average annual AI, while temperature had no effect (Figure 3a; Table S1). However, the variation associated with the climate scenarios differed markedly depending on the period of the year and the abscission trait considered. Wide ranges of DFD were found for bunches pollinated in March and October, that is, around 1.5 days between the most extreme scenarios (Figure 3b). Both RF and RI variations were negatively correlated with DFD predictions, while T was positively correlated in March and negatively correlated in October (Figure 3b; Table S1). For AI, the range of variation was wide throughout the year except in March and April. Variations in both RF and RI were strongly and consistently correlated with AI predictions, while T was negatively correlated in January, February, and December, but not in May, August, October, and November or positively correlated in June, July, and September depending on the month considered (Figure 3b; Table S1.).

Despite the contrasting effects of climate scenarios depending on the month, comparison of annual trends in the extreme scenarios with those in current scenarios, seasonal patterns of DFD and AI were still observed (Figure S10).

## 4 DISCUSSION

### 4.1 Coupling statistical and ecophysiological modeling is an efficient approach to analyze environmental regulation of plant development

The strong seasonal and interannual environmental variability found in the southern region of Benin where the present study took place offered a unique opportunity to test the effects of environmental variations on fruit abscission. Identifying the environmental determinism of traits of interest is an important research field in plant science, but standard methods and statistical approaches, such as single variable analysis, cannot address the complexity of environmental variations (Pol et al., 2016). Specific challenges concern the correlations between the environmental variables caused by climatic seasonality, which make it difficult to identify the true environmental signal, and the time lags between the signal and the phenotypic effect measured. In oil palm, the long fruit bunch development period generates such time lags, and the co‐occurrence of developing vegetative and reproductive structures produce feedback loops which can hamper studies on environmental regulation. What is more, the lack of information concerning the environmental regulation of fruit abscission, in particular in tropical perennial species, meant we were unable to test a mechanistic model based on a biological hypothesis. To overcome these limitations, we chose to use a group penalized approach. As demonstrated in genetic association studies (Ayers & Cordell, 2010; Huang et al., 2012), this type of approach outperforms single variable analysis by producing a sparse model containing the relevant groups of variables and the significant variables within those groups. Thus, in our study, with no a priori information, we were able to identify the best predictors among a set of environmental variables and specific times, simultaneously in a single model. While this method is well suited to select a single variable among a set of correlated variables, novel approaches that allow the selection of time periods instead of specific times are currently being developed for future studies.

Fruit abscission is coordinated concomitantly by environmental and endogenous regulatory systems (Sawicki et al., 2015). To take this into account, as putative predictors, we tested the calculated environmental variables (VPD or FTSW) and those related to carbon metabolism (DRD or SD). The calculation of these variables has been shown to have many advantages. First, computed environmental variables take plant development and growth processes into consideration and are closer to plant function and genetic determinisms than raw environmental variables (Chenu et al., 2009; Reymond, Muller, Leonardi, Charcosset, & Tardieu, 2003; Sinclair & Ludlow, 1986). Second, variables related to carbon metabolism take the internal carbon trophic state and the effects of plant ontogeny, development, and response to environmental constraints into consideration (Dingkuhn et al., 2007). In predictive and analytical approaches, the use of such variables can reduce the risk of over parametrization in purely additive models integrating various environmental effects (Luquet, Dingkuhn, Kim, Tambour, & Clément‐Vidal, 2006; Yan, Kang, Reffye, & Dingkuhn, 2004). Finally, these trophic variables, together with FTSW and TT, may be associated with cumulative variables, which do not depend on daily conditions alone and can thus provide an aggregated summary of one‐off records. Nevertheless, with the modeling approach, raw daily environmental variables (temperatures and solar radiation) can also be used to assess some environmental signals (Gyula, Schäfer, & Nagy, 2003; Penfield, 2008) that are not directly related to resource availability (water or carbon) or developmental processes (TT). By jointly selecting predictors among raw and calculated variables with the group penalized method, our approach enabled us to distinguish the contributions of different types of effects to the regulation of the fruit traits: the AI was mainly affected by environmental signals including temperature and solar radiation at specific stages, while DFD was also affected by carbon source/sink competition. Changes in carbon balance were previously observed to be associated with a number of reproductive traits, including BW (Combres et al., 2013;Pallas et al., 2013). Similar to those previous studies, in the present study we also find BW associated with carbon balance, which validates our approach (Figure S11).

We chose to use the group penalized method with environmental variable groups and the times within groups to identify sensitive stages during the inflorescence/bunch development. In the present study, we found a complex pattern of sensitive stages by considering combinations of trait and environmental variables. In previous studies using a correlative approach, Legros et al. (2009) and Pallas et al. (2013) found an inflorescence abortion sensitive stage between 11 and 13 months before harvest, corresponding to –150 to –186 DAP. Interestingly, in the same time frame, our approach revealed strong effects of solar radiation and temperature on BW (Figure S11), and VPD on DFD. In addition, those previous studies identified a sensitive period for the percentage of pollinated fruits around −35 DAP, which corresponds to the strongest effects of both the SD and temperature on BW in the present study. Taken together, it appears that developmental stages sensitive to environmental and endogenous signals at the bunch level may be shared, and that these co‐occurring processes could be co‐regulated.

### 4.2 Temperature perceived during specific stages of floral and fruit development modulates oil palm fruit abscission traits

The present study provides evidence that fruit abscission is a developmental process modulated by the environment, mainly by temperature, at specific stages of inflorescence/bunch development. The process of fruit abscission is tightly linked with plant development through the function of specialized AZ cells where cell separation occurs. Like in other fruit species, the oil palm AZ differentiates in the pre‐anthesis inflorescence before fruit development and only becomes functional later during fruit ripening (Henderson & Osborne, 1990; Roongsattham et al., 2012, 2016). The development of AZ is concomitant with floral development which occurs at −45 DAP based on the work of Adam et al. (2011). Interestingly, our data revealed that a high Tmin 45 days before pollination, reduced the competence of AZ to execute cell separation in fruit at 160 DAP. Modifications of the cell wall structure in the AZ earlier during development could possibly in turn modulate the timing of ripe fruit abscission. Indeed, modifications to the cell wall play key roles in plant adaptation to temperature changes and other responses to the environment (Ezquer, Salameh, Colombo, & Kalaitzis, 2020). Previous work revealed dynamic cellular and pectin structural modifications during oil palm AZ development and ripe fruit abscission that could be important factors for the timing of fruit abscission (Roongsattham et al., 2016).

Similar temperature effects on AI were observed at two specific stages of fruit development. The first stage at around 70 DAP includes the finalization of seed morphology when the endosperm becomes gelatinous, while the second stage at 150 DAP involves the peak of oil synthesis in the fruit mesocarp (Dussert et al., 2013; Tranbarger et al., 2011). These two sensitive stages are consistent with the waves of fruit abscission in the early stages observed in other fruit species, associated with the failure of embryo development or when competition for nutrients is high between vegetative organs (Sawicki et al., 2015). In the case of oil palm, abscission only occurs in the mature fruits, whereas the AZ may integrate these early perceived signals to modulate the intensity or the timing of abscission of ripe fruit. In contrast to the waves of fruit abscission during the early stages observed in other fruit species, high Tmin delayed the ripe fruit drop. At the ripe stage of oil palm fruit, a burst of ethylene is measured in the oil palm fruit (Tranbarger et al., 2011). In addition, exogenous ethylene treatments also induce abscission of oil palm fruit, but the effect is clearly enhanced in ripe fruit (Roongsattham et al., 2012). Ethylene production could be affected by temperature, as shown in other species (Antunes & Sfakiotakis, 2000; Field, 1985), which could have a cascade effect on the timing of fruit drop at the late stage of bunch ripening. In future studies, it would be interesting to investigate the ethylene levels in the AZ of ripe fruit subjected to contrasted temperatures.

Plant reproductive processes are more sensitive than vegetative growth processes to high temperature that can influence the productivity and adaptation of plants (Reddy, Hodges, & McKinion, 1997). While the physiological cause and molecular basis of fruit abscission of plants under high temperature conditions are largely unknown, different hypotheses can be evoked. For example, the supply of photosynthetic assimilates and plant hormone balance were found to be important in cotton and pepper fruit abscission respectively (Huberman, Riov, Aloni, & Goren, 1997; Zhao, Reddy, Kakani, Koti, & Gao, 2005). Concerning the relationship between plant hormone balance and abscission, the general concept is that as auxin is depleted across the AZ, ethylene sensitivity increases and acts as an abscission stimulator to change gene expression necessary for abscission to occur (Roberts et al., 2002). This is particularly true for senescing leaves or ripening fruit (Bangerth, 2000). A series of studies with chilling‐induced leaf abscission of *Ixora coccinea* plants found that although ethylene is essential for abscission, oxidative processes induced by the chilling stress are the trigger via a decrease in free indoleacetic acid (IAA) content and increased sensitivity to ethylene (Michaeli, Philosoph‐Hadas, & Riov j & Meir S., 1999). In fruit abscission, high night temperature stimulated drop of apple fruitlet and reduced their IAA export (Bangerth, 2000). Similarly, Huberman et al. (1997) suggested that the reduction of auxin transport capacity is the major mechanism by which high temperatures induce reproductive organ abscission in pepper. To gain insights into the mechanisms that govern all the main steps of abscission, it would be interesting to analyze the transcriptome, hormonal and metabolic reprogramming in the oil palm AZ during the key stages of floral and fruit development that are sensitive to this specific environmental signal identified.

Finally, temperature could affect components of the abscission signaling pathway to modulate abscission timing. For example, the peptide ligand‐receptor INFLORESCENCE DEFICIENT IN ABSCISSION (IDA)‐HAESA (HAE) and ‐HAESA‐LIKE2 (HSL2) pathway is a core signaling cascade involved in environmentally triggered abscission (Patharkar & Walker, 2016). Components of this pathway were identified in the oil palm AZ (Stø et al., 2015; Tranbarger et al., 2019) and could be targeted by temperature variation. Future work could focus on whether and how temperature may act to modify the function of core abscission pathway components.

### 4.3 Seasonal environmental variation and trophic status modulate the timing of oil palm fruit abscission more than extreme environmental scenarios

Our data reveal that seasonal changes in tropical regions have a strong influence on the oil palm ripe fruit abscission process. In oil palm, we found that environmentally stressful conditions generally delay abscission, as shown by both phenotypic analyses and climate simulation‐based prediction. In other species including citrus and olive, water conditions were previously shown to be a significant regulator of immature fruit abscission (Lavee, Nashef, Wodner, & Harshemesh, 1990; Mahouachi, Gómez‐Cadenas, Primo‐Millo, & Talon, 2005). In oil palm, there is no immature fruit abscission, but there are other adaptive traits affected by water conditions, including sex ratio and the abortion of female inflorescences (Pallas et al., 2013). Considering the marked alternation of rainy and dry periods in Benin, we expected to find an effect of water condition on mature fruit abscission. Surprisingly in the present study, temperature was found to be the main determinant of the timing of abscission. Our findings provide evidence that throughout inflorescence/bunch development, AZ cells act as sensor cells that respond to environmental signals to regulate the timing of cell separation in ripe fruits. For an appropriate and consistent response to seasonal changes, temperatures appear to be a robust environmental signal to the plant because they are less random and less subject to interannual variations than rainfall events. These results suggest similarities with the slight variations in photoperiod that regulate other developmental processes observed in different tropical species (Linnemann, 1991; Marcos, Cornet, Bussière, & Sierra, 2011).

The sensitivity analysis based on climate simulations revealed that seasonality is the major source of variation in the timing of oil palm fruit abscission, not extreme environmental scenarios. In our climate simulations, water conditions are predicted to affect fruit abscission, depending on the season considered and particularly during seasonal transitions. Moreover, water conditions have an impact on the number of bunches that develop (Oettli et al., 2018). This aspect was not investigated in the present study but could also affect the timing of fruit abscission indirectly through a change in the carbon balance. This indirect effect could be examined by including retroactions between climate conditions and plant development, for example for sex determination, inflorescence abortion or rate of inflorescence development as already done in mechanistic models (Combres et al., 2013). Importantly, in the present study, we observed that similar developmental periods during inflorescence/bunch development are sensitive to environmental signals that ultimately have an effect on both the timing of fruit abscission and other bunch production components, which in turn affect oil yield and the quality.

## CONFLICT OF INTEREST

The authors declare that the research was conducted in the absence of any commercial or financial relationships that could be construed as a potential conflict of interest.

## AUTHOR CONTRIBUTIONS

Design of the research: ST, TJT, and FM; Calculation of ecophysiological variables, statistical analysis and climate change stimulation: ST, MD, and BP; phenotype acquisition: ST, HD, MC, TJT, and FM; Acquisition of environmental data: HD and MC; Interpretation: ST, BP, and FM. ST, TJT, and FM wrote the article. All the authors read and approved the final manuscript. Project administration and funding: TJT and FM.

## Supporting information

Figure S1‐S11

Table S1
