# Supplementary material for: Environmental and trophic determinism of fruit abscission and outlook with climate change in tropical regions
Source: Plant Environ Interact. 2020 Apr 22;1(1):17–28. doi: 10.1002/pei3.10011 (PMC10168054; doi:10.1002/pei3.10011)

Figure S1. Pedigree of the oil palm population studied. All individuals in the population are derived from a single individual by successive self-fertilization. The number of individuals per progeny are indicated. The plants were between 10 and 15 years old at the start of the study.

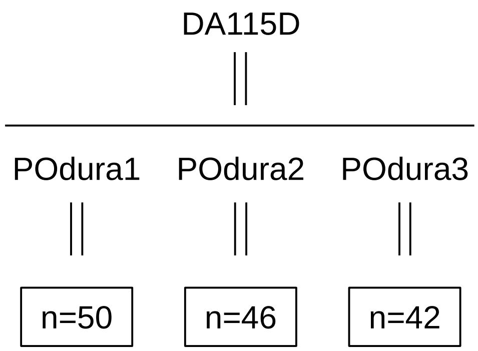

Figure S2. Distribution of the days to fruit drop in the oil palm population studied. The days to fruit drop were recorded on 4,835 bunches over a period of 12 years in the three progenies studied. The green line indicates the timing of the abscission in-vitro tests. DAP: days after pollination.

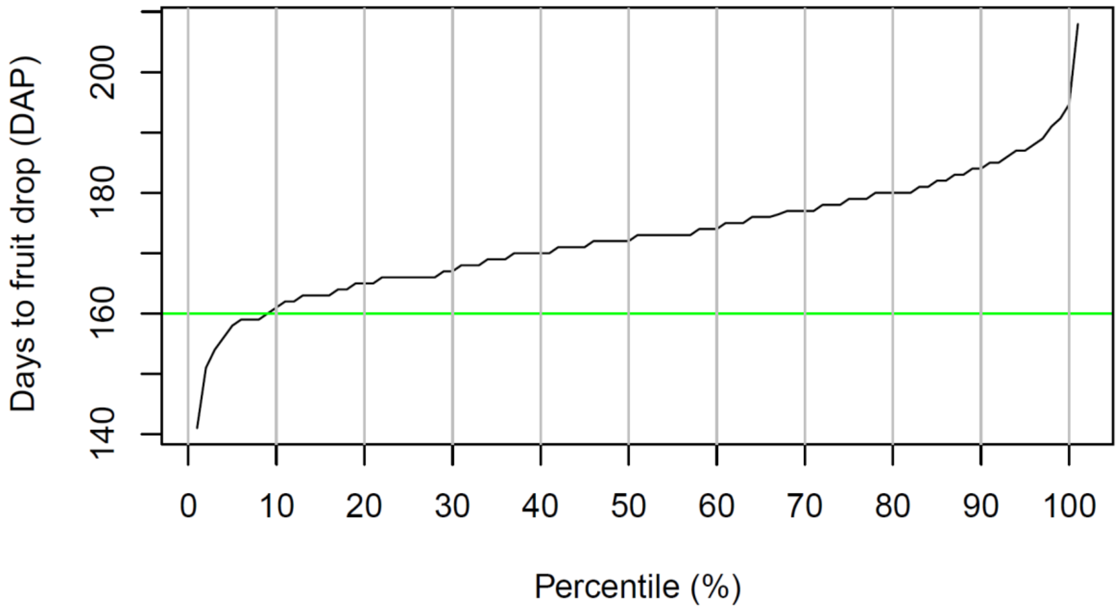

Figure S3. Attribution of classes. The four phenotypes defined for the test used to calculate the abscission index (AI). (A) no separation, (B) beginning to separate in the primary abscission zone (AZ), (C) separation in the primary AZ, (D) complete separation in both primary and adjacent AZs.

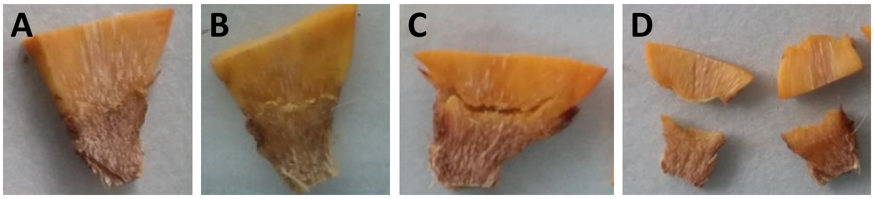

Figure S4. Computation of daily thermal time.

The formalism used to compute daily thermal (TT) was previously described in Combres et al. 2013 and is based on a trapozeoid response curve accounting for the effects of minimal and maximal daily temperature (TMin, TMax). This formalism integrates four parameters (Tbase, Topt1, Topt2, TLim) whose values were also determined in Combres et al. 2013. Tbase and TLim are respectively the temperatures below and above which no development occurs. The developmental rate is considered as maximal in a range of temperatures between Topt1 and Topt2. The model then assumes a linear increase in the developmental rate between Tbase and Topt1 and a linear decrease between Topt2 and Tlim.

The following code (R software) was used to compute the daily thermal time:

```
compute_daily_thermal_time = function(TMin, TMax, Tbase, Topt1, Topt2, TLim)
{
if (TMin < Topt1)
                {V = ( (min(Topt1 , T Max) + TMin) / 2 -Tbase) / (Topt1 -Tbase)}
else
{V = 0}
if (TMax > Topt2)
{W = (Tlim - (TMax + max(Topt2, TMin)) / 2) / (Tlim - Topt2)}
else
               {W = 0}
if (TMax < Topt1)
               {S2=0}
else {
               if (TMax < Topt2)
{S2 = TMax - max(Topt1, TMin)}
               else {
                              if (TMin > Topt2)
                                             {S2 = 0}
                              else
                                             {S2= Topt2 - max(Topt1, TMin)}
                    }
   }
m1 = V * (min(Topt1, TMax) - TMin)
m2 = W * (TMax - max(TMin, Topt2))
TT= ((m1 + m2 + S2) / (TMax - TMin)) * (Topt1 -Tbase)
return(TT)
}
```

Figure S5. Variation in environmental variables and in the days to fruit drop (DFD) in oil palm grown in Pobè, Benin. Monthly averaged environmental variables and the day to fruit drop are plotted for 12 recorded years. DFD was recorded on individuals derived from self-pollination of a single palm, and for individuals over six years old. DFD is plotted against the month of artificial pollination of the corresponding bunch. Each of the four contrasted seasons are indicated by shading, grey representing rainy seasons. Tmax, maximum temperature; Tmin, minimum temperature; RH, relative humidity; R: rainfall; SR: solar radiation.

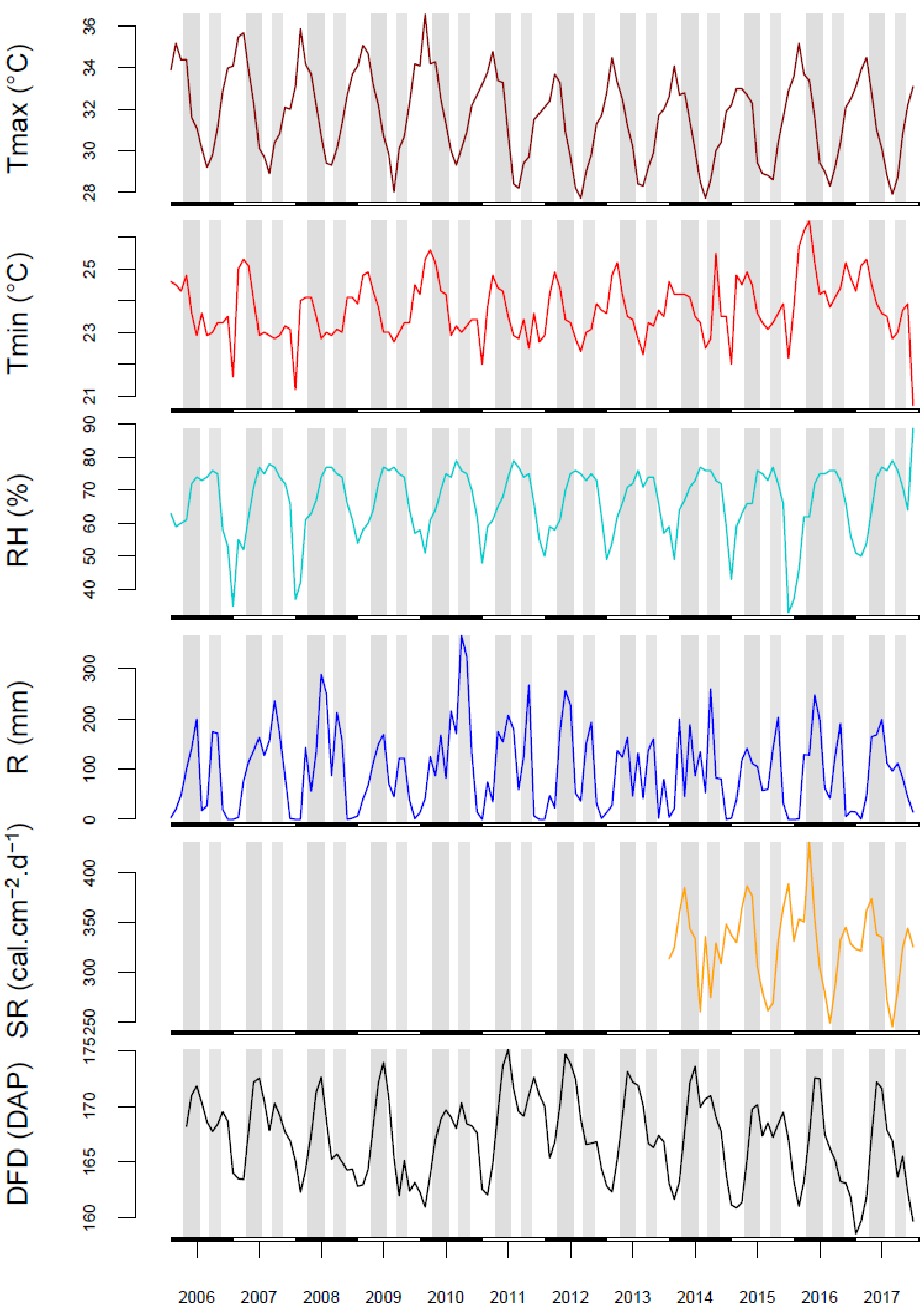

Figure S6. Seasonal variation in the abscission index (AI). Monthly average values of the AI are plotted against the month of expected fruit drop of the corresponding fruit bunch. Each of the four contrasted seasons are indicated by shading, grey representing the rainy seasons. Vertical bars represent standard deviations.

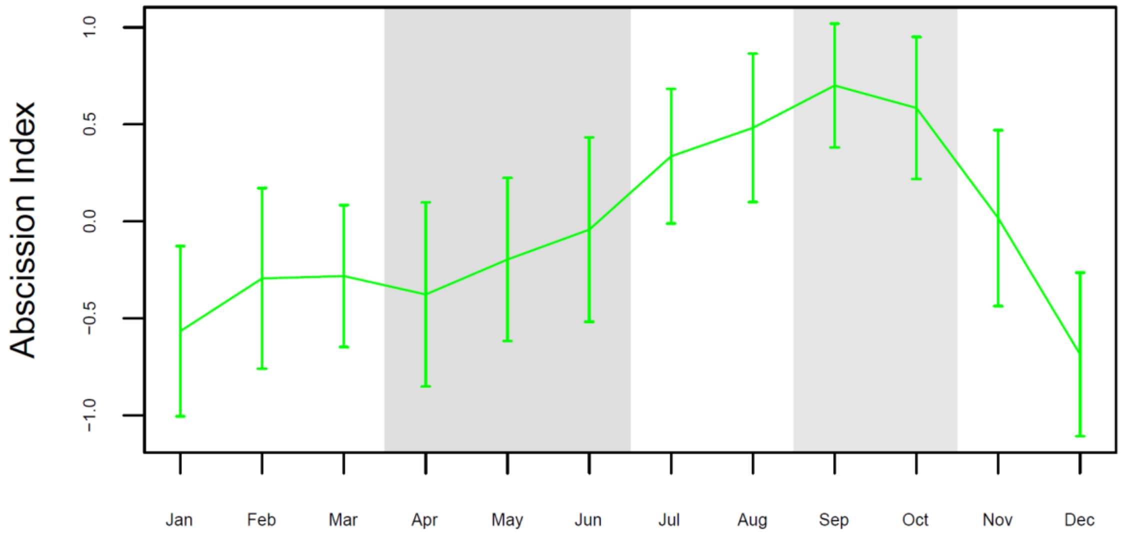

Figure S7. Environmental and trophic effects on the days to fruit drop (DFD) and on the abscission index (AI). Temporal profiles of p-value were obtained for each variable and each trait analyzed using univariate linear regression models. The logarithm of p-value were plotted with the sign indicating the sign of the regression coefficient, positive or negative. Shading corresponds to different p-value intervals. Known stage transitions during inflorescence/bunch development are indicated by dotted vertical lines on the corresponding days after pollination (DAP).

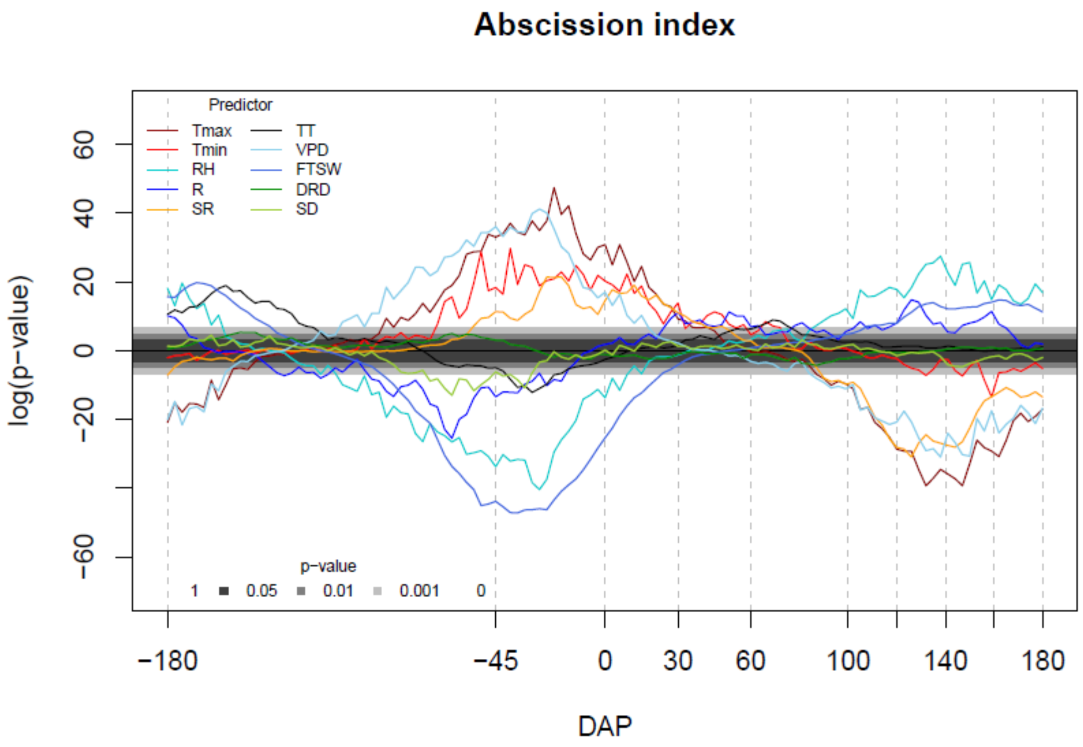

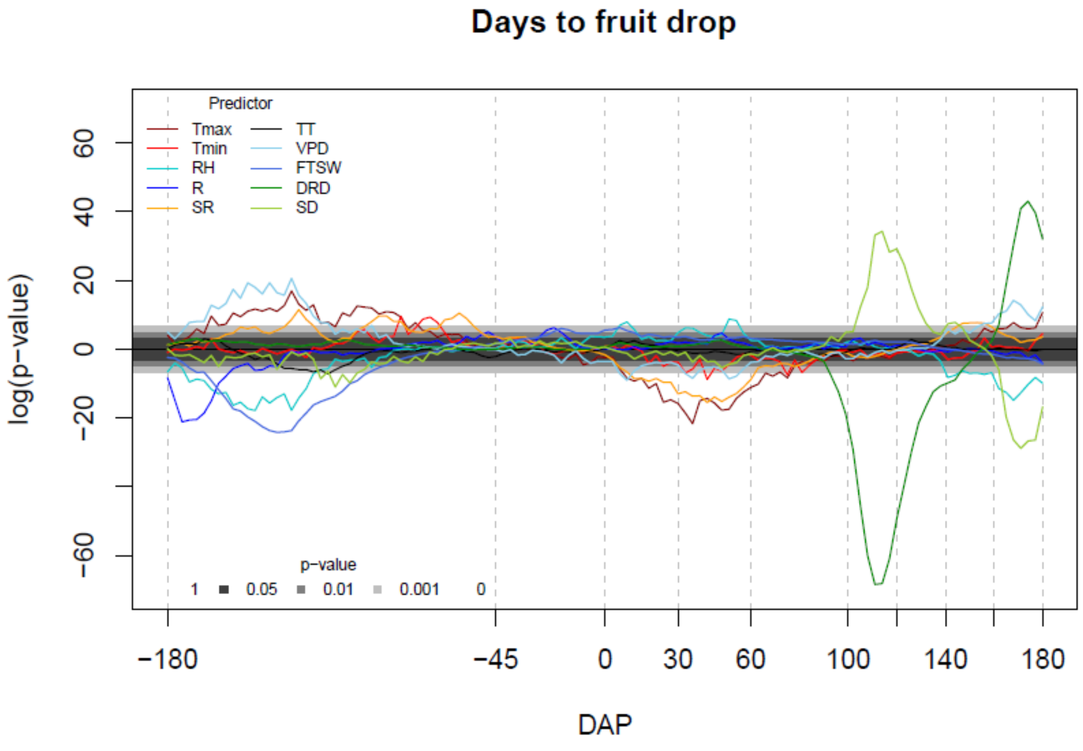

Figure S8. Effects of thermal time on the days to fruit drop (DFD) and on the abscission index (AI). (A) Relationship between the thermal time at the stage (160 DAP) of the in-vitro phenotype test and DFD (left panel) and the AI (right panel). The solid line represents the linear regression. (B) Coefficients obtained for 9 environmental/trophic variables using a penalized method plotted against the corresponding thermal time since pollination of the inflorescence. Tmax, maximum temperature; Tmin, minimum temperature; RH, relative humidity; R: rainfall; SR: solar radiation; VPD: maximum daily vapour pressure deficit, FTSW: fraction of transpirable soil water, DRD: daily reproductive demand, SD: supply-demand ratio.

A

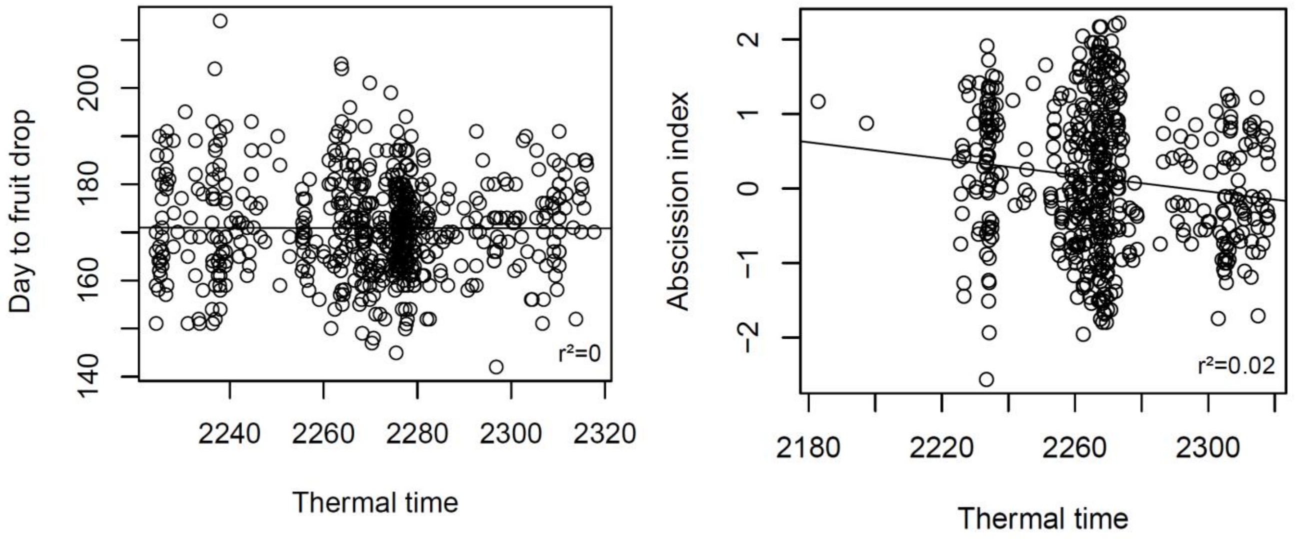

B

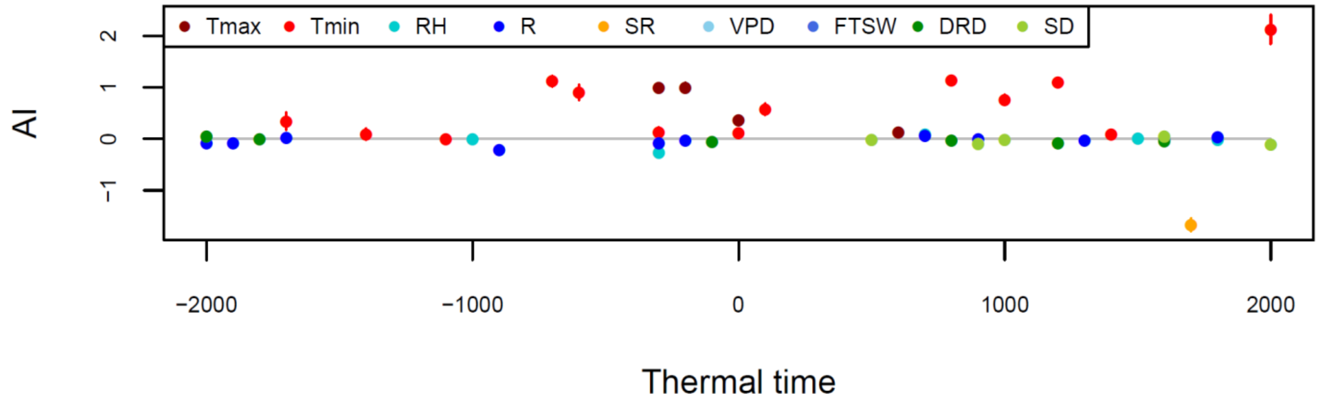

Figure S9. Model fitting quality measured by the correlation between observed and predicted fruit traits. For each fruit trait, the solid line represents the regression between observed and predicted values and r the correlation coefficient. In the top panel, dots are colored according to the three progenies studied (name of the self-pollinated parent is indicated), and in the bottom panel according to the month of the pollination. AI, abscission index; DFD: days to fruit drop; BW: bunch weight.

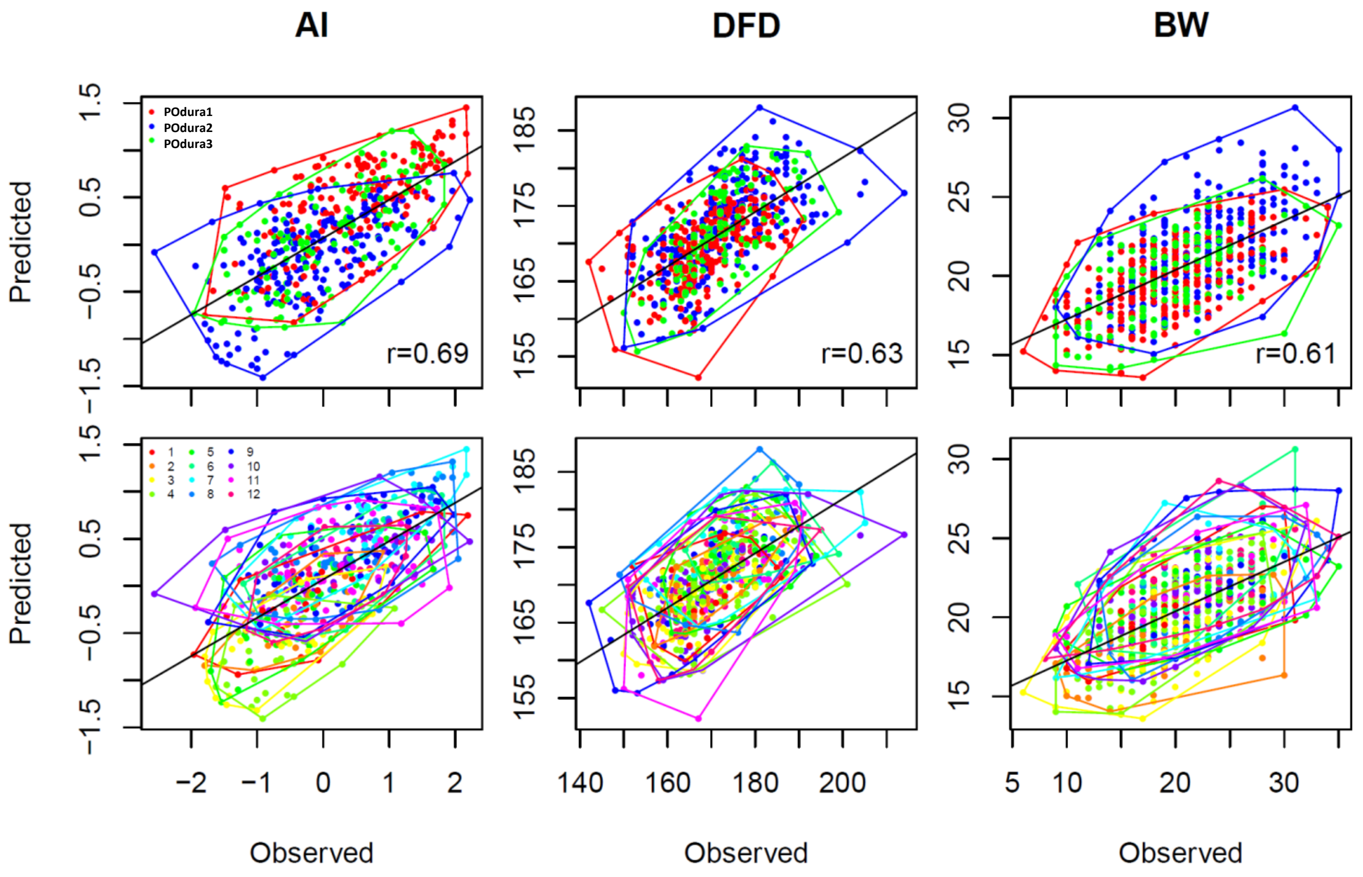

Figure S10. Prediction of the impact of climate scenarios on fruit abscission traits in oil palm. Monthly average predicted values of days to fruit drop (black) and of the abscission index (green) are plotted for current climatic scenarios and the scenarios with the lowest and highest annual predicted values of each trait.

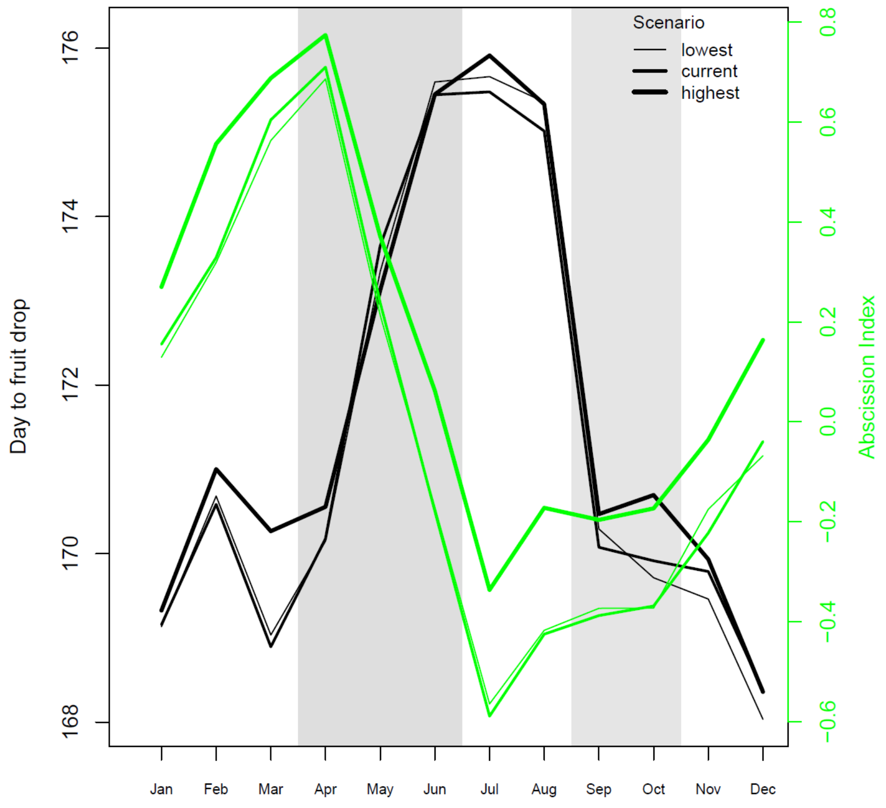

Figure S11. Analysis of seasonal bunch weight patterns and predicted impact of climate scenarios.

A. Monthly averaged bunch weight plotted for the 12 recorded years.

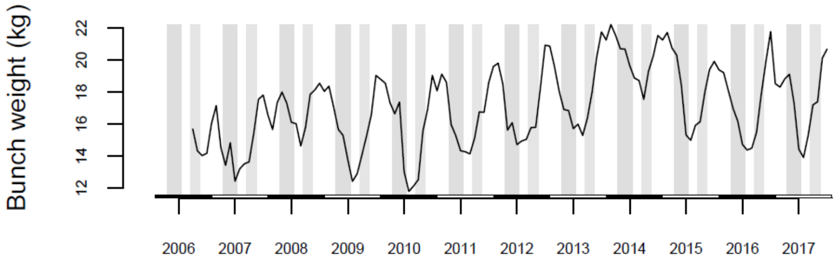

B. Environmental and trophic effects analyzed using univariate linear regression models (See legend to Fig. S7).

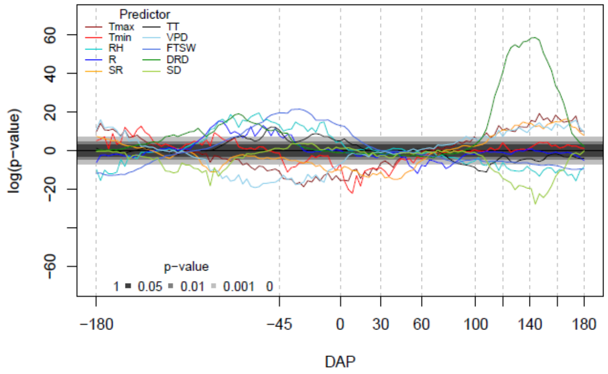

C. Cumulative coefficients (left) and patterns of coefficients (right) obtained for 9 environmental/trophic variables using a penalized method (see legend to Fig. 2).

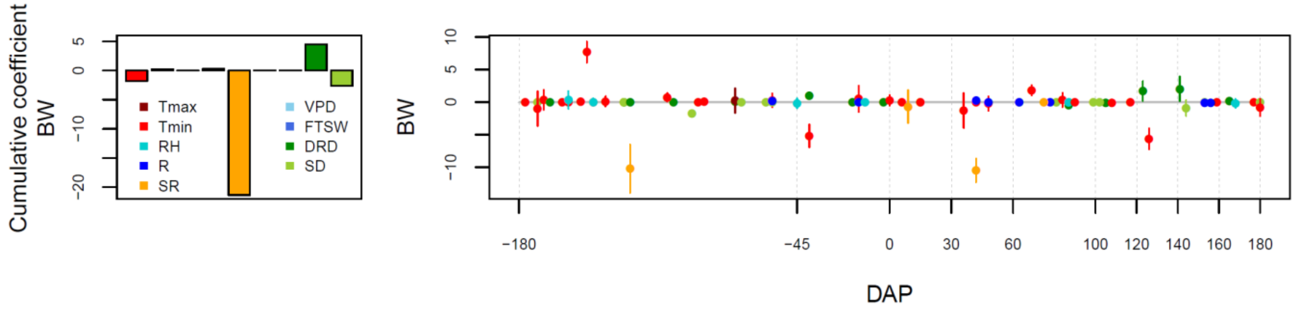

D. Annual (left) and monthly (right) average predicted values (see legend to Fig. 3).

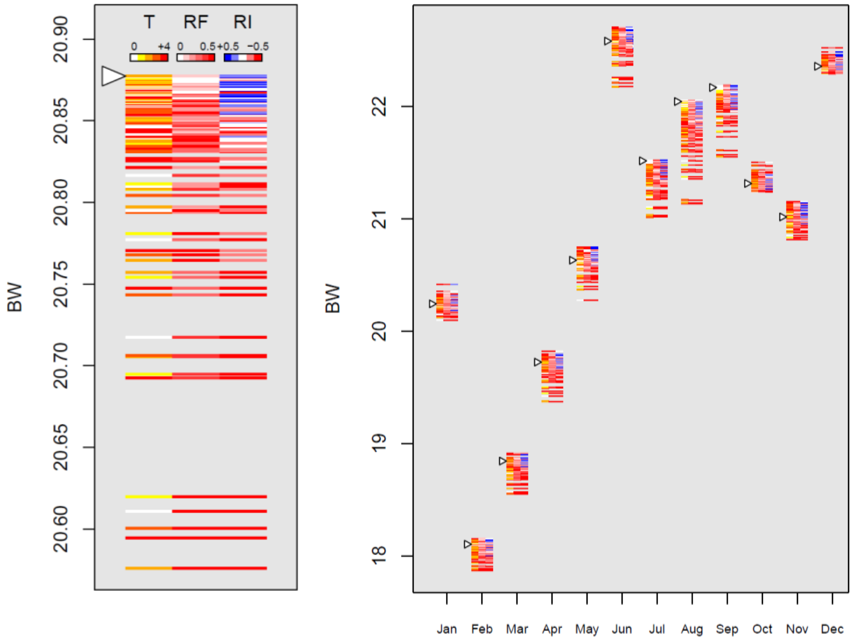

Supplement: Supplementary file 1 — Figure S1‐S11 [file PEI3-1-17-s001.pdf]
