# Supplementary material for: Environmental and trophic determinism of fruit abscission and outlook with climate change in tropical regions
Source: Plant Environ Interact. 2020 Apr 22;1(1):17–28. doi: 10.1002/pei3.10011 (PMC10168054; doi:10.1002/pei3.10011)
Supplement: Supplementary file 2 — Table S1 [file PEI3-1-17-s002.pdf]

Table S1. Correlations between the simulations of climate parameters and the predicted values of the fruit traits. Spearman correlation coefficients (ρ) were computed between the predicted values of the day to fruit drop (DFD), the abscission index (AI) and the bunch weight (BW) against temperature (T), rainfall frequency (RF) and intensity (RI) simulated in the different climate scenarios tested. Coefficients are presented with annually or monthly averaged predicted values of fruit traits.

Trait	Env	ρ_Year	ρ_Jan	ρ_Feb	ρ_Mar	ρ_Apr	ρ_May	ρ_Jun	ρ_Jul	ρ_Aug	ρ_Sep	ρ_Oct	ρ_Nov	ρ_Dec
DFD	T	0.33***	0.26**	0.49***	0.45***	-0.05	-0.92***	0.58***	0.90***	0.92***	0.54***	-0.48***	-0.79***	-0.71***
	RF	-0.39***	0.10	-0.16	-0.48***	-0.22**	0.07	0.13	-0.07	0.02	-0.26**	-0.27**	-0.15*	-0.11
	RI	-0.36***	0.39***	-0.21*	-0.46***	-0.42***	-0.04	0.20*	-0.04	0.08	-0.25**	-0.26**	-0.17*	-0.15
AI	T	-0.12	-0.41***	-0.22**	-0.38***	-0.42***	-0.12	0.38***	0.24**	0.08	0.31**	-0.04	0.13	-0.45***
	RF	-0.66***	-0.49***	-0.48***	-0.35***	-0.33***	-0.43***	-0.40***	-0.52***	-0.58***	-0.40***	-0.46***	-0.42***	-0.45***
	RI	-0.65***	-0.47***	-0.52***	-0.45***	-0.32**	-0.53***	-0.50***	-0.49***	-0.54***	-0.58***	-0.58***	-0.56***	-0.47***
BW	T	-0.20*	0.10	-0.31**	0.03	0.20	0.39***	0.34***	-0.50***	-0.52***	-0.66***	0.31**	0.34***	0.43***
	RF	0.62	0.22**	0.37***	0.43***	0.42***	0.30**	0.48***	0.35***	0.38***	0.36***	0.04	0.34***	0.20*
	RI	0.62***	0.12	0.35***	0.54***	0.46***	0.38***	0.45***	0.49***	0.51***	0.39***	0.01	0.42***	0.23**
